# On the Teaching of Science, Technology and International Affairs

**DOI:** 10.1007/s11024-012-9191-9

**Published:** 2012-02-11

**Authors:** Charles Weiss

**Affiliations:** School of Foreign Service, Georgetown University, 37th and O Sts., N.W., Washington, DC 20057 USA

**Keywords:** Science policy, Innovation policy, Undergraduate education, University curriculum, Environment, Security, Energy, Development, Health, Georgetown University

## Abstract

Despite the ubiquity and critical importance of science and technology in international affairs, their role receives insufficient attention in traditional international relations curricula. There is little literature on how the relations between science, technology, economics, politics, law and culture should be taught in an international context. Since it is impossible even for scientists to master all the branches of natural science and engineering that affect public policy, the learning goals of students whose primary training is in the social sciences should be to get some grounding in the natural sciences or engineering, to master basic policy skills, to understand the basic concepts that link science and technology to their broader context, and to gain a respect for the scientific and technological dimensions of the broader issues they are addressing. They also need to cultivate a fearless determination to master what they need to know in order to address policy issues, an open-minded but skeptical attitude towards the views of dueling experts, regardless of whether they agree with their politics, and (for American students) a world-view that goes beyond a strictly U.S. perspective on international events. The Georgetown University program in Science, Technology and International Affairs (STIA) is a unique, multi-disciplinary undergraduate liberal arts program that embodies this approach and could be an example that other institutions of higher learning might adapt to their own requirements.

Science and technology are critical dimensions of many if not most of the critical issues of international relations: competitiveness, nuclear proliferation, terrorism, Internet governance, renewable energy, cybersecurity, asymmetric warfare, nuclear and offshore drilling accidents, space, genetically modified crops, human cloning, synthetic biology, epidemic disease, climate change and many more. Each of these issues requires attention to the interface of science and technology with economics, politics, law and culture – an interface that lies at the heart of science and technology policy. These issues affect organizations as different as Google, Pfizer, BP, Monsanto, and the Inter-Governmental Panel on Climate Change (IPCC), the Ventner Foundation, the Archives of Internal Medicine, the Council on Competitiveness, and the U.S. Department of State.

The increasing importance of science and technology in policy issues has focused increased attention on the teaching of science and technology policy, both to prospective or mid-career scientists, doctors and engineers, on the one hand, and to generalist policymakers, entrepreneurs, business managers and other professionals, on the other. Even so, the discipline of international relations is responding only slowly to this critical need. A few graduate and undergraduate programs have sprung up to meet the demand for such training, but there is little literature as to how this subject should be addressed in an academic setting.

University curricula in international science and technology policy are fated to address an impossibly varied menu of topics. Just the limited list in our opening paragraph covers a broader range of topics than are dealt with in a typical multi-disciplinary curriculum – say, in political economy or even in environmental science. To add to the complications, the disciplines involved in STIA encompass essentially all branches of the natural sciences and engineering, plus all of the social sciences and much of the law, with a bit of philosophy and other humanities thrown in. Some of the issues are domestic, others cross borders, and still others are global. Some require international comparisons, and some (like energy, environment and epidemiology) are “intermestic” in the sense that they involve seemingly purely domestic policies that have important international or global ramifications.

What can possibly hold together such a disparate set of topics and disciplines? How do we take students from their starting point – typically an undergraduate degree in a single discipline in natural or social science or engineering – and give them a feel for the myriad influences that can affect the progress of science or technology and the impact of that progress on the broader society?

The good news is that this diversity of background may not matter too much, at least not for our purposes as educators. Experience has shown that wherever a student starts to address almost any of these topics, (s)he is likely to trace out a web of interconnections among science, innovation, economics, business, politics, and ethical and philosophical values that is enough to give students a feel for the basic patterns that apply to almost any topic in science and technology policy.^1^ After all, specialists in science and technology policy have come to the field from any of a wide variety of backgrounds: natural science, engineering, political science, economics, sociology, linguistics, law, journalism and many others.

At least in my generation, almost all of these policy practitioners and academics backed into the field at a point in their careers when they realized that the individual discipline in which they were trained had not addressed aspects of great importance to the problems to which they were to devote their professional lives. Engineers, for example, typically realized that the achievement of their technical objectives frequently depended as much on economics, politics and public attitudes as on the quality of their technical concepts and designs. This is even more the case to the extent to which they conceive their task as one of designing and building a social space in addition to a physical construction. Scientists, for their part, often became concerned with the social consequences of the practical applications of their discoveries, and have confronted questions of ethics, politics and economics with which their education had not equipped them to deal. On the other side of the “two-culture” divide, social scientists, businessmen, and journalists often realized that their training – and indeed their socialization – had too often neglected the scientific and technological dimension of the issues with which they were dealing.

## Lessons and Meta-Lessons

This still leaves the question: Given the impossibility of teaching the entirety of the natural and social sciences in the year or two of courses to which we expose our students, what knowledge and analytic skills should we be imparting? First come the standard tools of policy analysis: cost/benefit and risk analysis, quantitative estimation, statistics, budgets, and the like. Second come broad conceptual frameworks useful in all fields in which science, technology and international affairs intersect: the politics, economics and management of innovation, the sociology of science, and the co-evolution of technology and society. Third and even more important comes discussion of values like equity, sustainability and privacy. These constitute the heart of science and technology policy – which is, after all, a branch of policy, not of science or engineering. Finally come the concepts, issues and disciplinary culture pertaining to specific areas of study, with proper attention to their scientific and technological dimension.

Over and above this substantive content, some of the most important dimensions of education in science and technology policy consist of philosophical or meta-lessons that have more to do with the general approach than with specific subject matter. For students whose primary training is in the social sciences, the first and foremost of these broad meta-lessons is the simple *importance and ubiquity* of science and technology as a dimension of international affairs. Its importance to environment, globalization, energy, information, competitiveness, space, terrorism, and military security should be obvious, although this is not always the case. The relevance of forensic anthropology and space-based remote sensing to human rights, or that of cybersecurity to international finance may be less obvious, but is no less important.

A second meta-lesson is the need for policymakers or scholars to *overcome their fear* of science and technology and to respect and master technical material, seeking sources and asking questions until they understand it well enough to pose the broader policy questions, whether or not this technical material comes from a discipline with which they are comfortable. With very few exceptions, technical material understandable to the non-specialist is available on any topic of policy interest, and experts are available to help policy practitioners over the rough spots.

As a natural scientist, I instinctively feel that the more training in natural science a student brings to a subject, the better he or she will be able to deal with the most conceptually difficult aspects, namely those that involve “real” science. However, this preconception is not always borne out by experience. Many of the most acute critics of science and technology have little formal technical training, but have learned what they need to know.

Here the key point is to respect and master the lessons drawn from disciplines other than your own. At the same time, it is essential to remember that different disciplines have different methods, terminology, models, types of evidence, and standards of proof. Equally important, they have different attitudes, traditions, sociology and culture. Many ecologists and environmentalists, for example, see all of Nature as a seamless web, and hence tend toward precaution – whereas engineers and the more reductionist scientists (molecular biologists or physicists, for example) tend, with important exceptions, to be more compartmentalized in their view. Here the historical, sociological and philosophical insights provided by our colleagues in “science and technology studies” (STS) are valuable.

The third meta-lesson is to cultivate both an *open mind and a fearless, skeptical and critical attitude* – to keep your hand on your wallet, to quote the traditional warning in places frequented by pickpockets - so as to be able to distinguish between issues of perception and those with a real basis in science. One of the most important skills of the policymaker is to be able to sort out disagreements among experts on a subject of which (s)he may have only a limited initial understanding. This means ensuring that the arguments on the various sides of the issue are consistent with what is known of the science, and with the present and likely future capabilities and limitations of the technology, and that they take into account the uncertainties in the current state of scientific knowledge, and the even greater uncertainties in the prediction of the likely impact of any given technology.

At the same time, it is likely that much essential information in such a situation will come from parties that may have hidden assumptions, that may not understand the broader issues, or that may have a vested interest in a given interpretation of current knowledge. Here it is important to examine with an open yet critical mind scientific information that comes from a source that is politically distasteful or that has an interest in a particular outcome, and to resist the temptation to trust only those sources that share your political orientation.

A fourth meta-lesson, one particularly important in international work, is to maintain a global overview – or to say it another way, to avoid adopting a purely American outlook, or worse, allowing the policy agenda of the U.S. government to dominate one’s academic pursuits.

For science students getting their first exposure to policy matters, the meta-lessons are somewhat different but no less important. Here the objective is to increase the students’ skill and comfort level in dealing with the sociology of science, the relation of scientists with the political system, and the economic, political and cultural factors that influence science and technology and their impact on the larger society. Perhaps most important, these students need to learn to present the scientific dimension of broader issues clearly and accurately to a non-scientific audience, to explain and put into context the inevitable uncertainties and areas of ignorance, and to appreciate the fact that public attitudes toward science- and technology-intensive issues are influenced by value systems that may be different from their own.

## The Science, Technology and International Affairs (STIA) Program at Georgetown University

Against this background, it may be useful to describe the undergraduate liberal arts program in science, technology and international affairs (STIA) of the Georgetown University School of Foreign Service (SFS). This is the program in which I have taught for fifteen years. It offers an example of how to approach the teaching of this vast and sprawling subject. STIA is one of seven majors of the School, and attracts 45-60 majors each year out of a cohort of about 325, plus a few minors from the Georgetown College who typically are pre-medical students who want to broaden their understanding of policy matters. As far as I know, the scope of this undergraduate program is unique in the United States.

Our students come to us after completing “social science boot camp”: two years of economics, a year and a half of international relations, a year and a half of world history, a year of philosophy and theology, and the pathway to a high level of foreign language proficiency. This sequence of courses conveys a good feel for the inter-relations among politics, economics and culture, and constitutes excellent preparation for multi-disciplinary study. By graduation, most of our students have only two terms of natural science designed for science majors, and a term each of college-level math and computer science – about the equivalent of the first semester of a good science degree. This is considered a lot of science in the rather technophobic culture of international relations students; in fact, STIA majors are still the only SFS students who are required to take any science courses at all, although this is likely to change soon.

The STIA major is divided into four concentrations: energy and environment, security, international health, and business, growth and development. Each of these explores the mutual interaction of science, technology and international relations in its particular sphere. The emphasis here is on the word “mutual”: science and technology influence international relations, and international relations influence science and technology – in short, they “co-evolve.” STIA courses are generally expected to consist of about 30-40% technical material on natural science (or technology based on natural science), and the rest on the surrounding economic, business, political, legal, philosophical and cultural context.

For these purposes, technology is defined simply as a way of solving a practical problem through the use of technical knowledge. As such, information and communication technology is considered to be a branch of technology on an equal plane with technologies based on the more traditional biological, chemical, geological and physical sciences. We also consider technologies at all levels of sophistication – from relatively advanced nuclear, nano- or biotechnology to more basic areas like water supply and sanitation. Both high and low technologies may have major effects on human welfare, both positive and negative. For example, and sad to say, two simple technologies – the truck bomb and crack cocaine – are among the most important technological innovations of the past few decades.

### Introduction to STIA

I developed and teach the four-credit introductory course, the only course required of all STIA students and one widely viewed as a demanding rite de passage. The course is a survey of the interactions among science, technology, economics, business, politics, law and culture. It provides a brief introduction to each of the concentrations, and illustrates the themes set forth in the earlier paragraphs of this essay. It also does its best to encompass the meta-lessons we discussed earlier.

The course begins with some general observations regarding how scientific knowledge advances, and how this advance – and the uncertainties inevitably associated with that advance – affect international diplomacy, using the international negotiations over climate change and the destruction of the stratospheric ozone layer as case studies. It then explores the management of technological innovation at the firm level, motivating student interest on the grounds that technological innovation in environment, security, energy and health (which are the more immediate interests of most of our students since the dotcom crash) is likely to take place in firms.

The course continues with a quick introduction to the economics of technological innovation, including a discussion of productivity and international competitiveness and the principles underlying national science and technology policy, and a brief survey of the justification, objectives and instruments of science and technology policy, the institutions and other actors that are involved, and the mechanisms by which scientific advice enters the policy process. It then briefly explores the role of science and technology in environment, biotechnology, health and epidemic disease, energy, space, information and communications technology, e-commerce, anti-poverty technology for developing countries, and security (including conventional military strategy, nuclear proliferation, information warfare, terrorism and homeland security), in each case touching on both business and public policy issues.

The course concludes with a brief comparative survey of national innovation systems in the U.S., Europe, Japan, Korea, India and China, along with general comments on the technological development of developing and countries in transition to market economies. For this purpose, we consider the national innovation system to include not only the institutions and policies directly concerned with science and technology, but also the broader economic, political, social and cultural factors bearing on science, technology, and the impact of innovation. For example, the course stresses that the strength of the U.S. in innovation stems not only from its large research budget and elite educational institutions of higher learning, but also from a culture that exalts innovation and accepts failure as an inevitable element of the innovative process.

We try hard not to yield to the temptation of American triumphalism, not only because of the current financial crisis and Great Recession, but more fundamentally because there are important areas in which the U.S. is hardly a technological leader. Many of the most important of these are in economic sectors dominated by well defended, entrenched legacy technologies guarded by politically powerful vested interests. Fossil fuel-based energy, the electric grid, health delivery, and transportation are prominent examples. Innovation theorists tend to neglect these areas in favor of more exciting technologies like nano- and biotechnology, but they are critical elements of future economic competitiveness and social equity and deserve more focus on the part of the science and technology policy community.

Students tell me that the course has a certain resemblance to drinking from a fire hose, but that it introduces them to a broad range of concepts that are useful later when they drill down into their particular areas of concentration. The course readings stress the interactions of science and technology with the broader context, but do not shy away from explicit consideration of technical material. The initial assignments of the course are designed, first, to overcome the technophobia endemic in the international relations profession, second to instill confidence that students can master scientific topics if they put their minds to it, and third to give students some practice in using their own judgment in addressing disagreements among technical experts. The 15-page term paper may be on any topic of the student’s choice that combines science and technology with broader considerations, but must include a section that sets forth the relevant technical material in language that is at the same time precise, accurate, and accessible to the educated lay person.

Our students benefit greatly from strong programs in other parts of the university in global health, Internet policy, and security and peace studies. They also benefit from the fact that Washington, in common with some other university cities, is full of capable and experienced expert practitioners who are willing and often eager to give courses in their specialty as adjunct faculty for essentially pro bono wages (although Georgetown does pay its adjuncts more than most other local universities). Washington is also a rich locus of policy-oriented internships, opportunities that our students assiduously pursue and that greatly enrich their experience. STIA faculty work within the Washington policy community and help students identify internships and get placed in interesting positions.

### The STIA Concentrations

The remaining courses taken by STIA majors are more specific to the individual concentrations and reflect and explore their differing combinations of domestic and international science, technology, economics, politics and culture, as well as their different time horizons. Each of the four full-time faculty members in the STIA program offers courses in fields related to their own research. These courses and this research is in each case multi- rather than inter-disciplinary; in other words, the integration of multiple disciplines takes place in the mind of an individual researcher or instructor, as opposed to a collaboration of scholars and instructors trained in different individual disciplines. Energy in India and China, technology in military and civilian intelligence, technology and poverty in developing countries, oceans and policy, and the politics of international health are examples.

Energy and environment, the first and currently the most popular STIA concentration, provides perhaps the clearest examples of this complex mix. As in other universities, our courses in environmental science study how the earth’s systems interact and how humans affect them. But this is only the beginning. The ultimate cause of any problems that are found often lies in policy – typically a failure to effect or enforce environmental policy or to charge users for some important environmental resource or service. Behind the policy failure lies still another “ultimate” cause, namely domestic or international politics, and behind this often lie cultural attitudes toward the priority of environment as compared to economic growth or other desiderata.

Energy science is also a science. Students of energy must understand basic scientific principles if they are to understand the economic and environmental tradeoffs among the many competing energy resources and technologies. Today, fossil fuels provide cheap and convenient energy – although at severe macroeconomic, environmental and geopolitical costs that are not borne by the user. Alternative sources – whether solar, wind, nuclear, or sources yet to be developed – must overcome a deeply entrenched techno-economic paradigm, an issue that raises a variety of inter-twined, high-stakes technological, economic, political, environmental, cultural and ethical issues that will be very difficult to resolve. The rapid growth of China and India adds an important new dimension, as both countries are not only major consumers of energy and major emitters of greenhouse gases, but also new aspirants to the role of world-class economic competitors and technological innovators.

The second STIA concentration, global health, operates at many levels. The study of the health of individuals requires knowledge of the natural sciences, especially biology and chemistry. The understanding of the dynamics of epidemic disease involves these sciences as well, but also mathematical statistics, sociology, and the domestic and international politics that determine the availability of health services and the ability and willingness of governments to gather and share information and of public health authorities to analyze and apply it. Even these complications yield too simple a picture. The health of the population of the world or any part of it depends on virtually every variable that affects overall well-being: income, employment, sanitation, environment, nutrition, personal behavior, education, housing, family sociology, gender, political stability, and so on – and last and not necessarily the most important, the availability of health services. STIA health courses, many provided by SFS’ sister School of Nursing and Health Studies, address these complications.

The technological aspects of security, the third STIA concentration, are best characterized as a complex mix of political and military strategy, and technology and technology management, along with a healthy dose of electoral and bureaucratic politics. Here an overarching theme is the constant competition between offense and defense, punctuated by technological revolutions that in turn stimulate counter-measures that overcome advantages of the new capabilities that had once seemed decisive. STIA courses explore this and other themes as they apply to the technological and political aspects of terrorism, intelligence, and nuclear proliferation, as well as conventional military strategy. Here we benefit from collaboration with Georgetown’s graduate program in Peace and Security Studies.

The fourth STIA concentration, known as Business, Growth and Development (BGD), has the largest emphasis on the discipline of international economics. Its focus is on technology management in international business and the technological dimension of both international competition and international development in the new global knowledge-based economy. It treats the policies by which different countries have stimulated technological capacity and innovation, with special emphasis on the United States and Finland and Japan in the advanced countries, and on Korea, Brazil, India and China among the so-called emerging markets.

For example, in the STIA course on Technology, Globalization and Growth, students learn about the role of technology in global economic development, and compare how different countries and regions have made effective use of technology to improve their growth and welfare. They then develop future scenarios that explore how different global drivers combining technological developments, economics and geopolitical forces may affect the relative performance of different regions and overall global welfare.

The BGD concentration puts special emphasis on information policy and the special problems posed by the governance of the Internet. Here the rise of India and especially of China raises issues, not only of language but also of the values of free expression and innovation that have hitherto been embedded in the network architecture but that now must be renegotiated as the Internet expands beyond its originally foreseen technical limits.

### Honors in STIA

Each year, four or five fourth-year students elect to write an honors thesis that delves deeply into a specific topic that links science and technology with its broader context. This is typically a 70-80-page document and takes a full year to complete. By and large, the research methodology is that of one or another of the social sciences, but the attention to the substance of science and technology goes well beyond that of most social science research.

The range of topics these students select for research is remarkably broad, and reflects the ubiquity of science and technology in contemporary international affairs. To cite a few representative examples, recent honors students have researched surrogate parenthood in India, the policy requirements of advanced geothermal energy in different countries, the health hazards of skin-lightening agents, and the access of North African immigrants to health services in Italy. The students involved in the latter two projects continued with related work after graduation, the first as a Fulbright fellow, the second as a volunteer in a non-governmental organization. Still other STIA honors students have won Fulbright fellowships for the study of the adjustments of East African farmers to climate change, the attitudes of rural Egyptian teenagers toward smoking, and multi-country surveys of different forms of solar, wind and geothermal energy. Student theses have resulted in publications in peer-reviewed journals in disciplines ranging from paleobotany to parasitology to South-East Asian studies to hydrology.

In several cases, the results of STIA honors research have led directly to practical application. For example, the conclusions of a STIA student’s review of the effectiveness of a USAID project for the use of cellphones for the coordination of logistical services in the health delivery system in Rwanda were directly incorporated into plans for a similar system in Tanzania. Another student’s research on Internet use in rural India led directly to a job developing information in local Indian languages in a form that would be useful to Indian farmers, as a complement to the development of innovative ruggedized and simplified Internet kiosks adapted to the harsh conditions of the Indian village.

On graduation, most of our alumni and alumnae launch much the same career patterns as other SFS graduates: law, business, intelligence, consulting, and political advocacy groups, with the exception that a few go on to medicine or science, including geosciences and physics. A minority become US Foreign Service officers. (SFS is the largest single source of Foreign Service officers, but these constitute only a small minority of SFS graduates, the name of the school notwithstanding.) The multi-disciplinary training of STIA graduates and their willingness to tackle science- and technology-intensive problems is well appreciated in government and in Washington-oriented consulting firms – although students sometimes report problems, as with any inter-disciplinary major, explaining to prospective employers exactly what STIA means.

Some of our graduates go on to an inspiring variety of other professions – from Secret Service agent to Microsoft executive to solar energy entrepreneur to patent lawyer to editor of software manuals. In the last four years, STIA graduates have won six Fulbright Scholarships, one Rhodes Scholarship, and four Circumnavigators awards – a Rotary Club award of a round-the-world study tour. One of our graduates planned the anti-HIV/AIDS campaign of a Caribbean Island while a Peace Corps volunteer and is now launching an NGO for health microgrants in developing countries. Two are advising municipal governments on green city planning, one in Sacramento and one in Melbourne, Australia. On the scientific side, one graduate has become a pioneering dendochronologist, applying techniques once thought limited to temperate deserts to the reconstruction of a two thousand-year history of the intensity of the tropical monsoon. Oh, to be twenty years old again.

## STIA and Science and Technology Studies (STS)

In sum, the STIA program is an internationally oriented, policy-motivated liberal arts program that explores the broad mutual influence of science, technology, economics, politics and culture, and how these influence and have influenced the policy process in an international context. Its scope is unique in its breadth. It explores the commonalities of a broad range of technologies, from environment and energy to health, information, security, and industry. Its focus is on economics, politics, area studies and language, with a good deal less attention to natural science than would be provided, for example, by a typical undergraduate major in environmental studies.

STIA draws on the ideas and concepts developed by the science, technology and society (STS) and science policy studies (SPS) programs found in several leading universities in the U.S. and abroad, but the emphasis is rather different. STS is a process-oriented branch of social science that addresses the workings of science and technology and their relationship with politics and the broader society as a social, anthropological, historical and epistemological system. SPS is a branch of policy studies with emphasis on the design of policies for research and innovation, the processes by which these are designed and implemented, and the effectiveness of these policies in achieving their stated objective, as well as their intended and unintended social impact.

STIA overlaps with each of these, but is more concerned with the interaction between science, technology and international affairs, and the practical outcome of this interaction. Happily, the three disciplines seem to be drawing closer together, so that the contrasts among the three approaches are not as sharp as they once were. Both STS and SPS are increasingly involved in international issues as these become more and more prominent.

I write this article to elucidate the Georgetown program and to make the case that other institutions of higher learning may well find it attractive to develop similar curricula, suitably adapted to their locale, their educational philosophy and approach, and their student body. More generally, STIA can be a vehicle to excite interest in science among non-science majors, and conversely to broaden the horizons of science students to encompass the social, political and economic context of their work and its potential impact on the larger society.

To the university faculty, STIA is an excellent nucleus for the integration of research and teaching in disciplines that span the full range of the natural and social sciences, and many of the humanities as well. To cite only two examples, poets, artists, philosophers and theologians need an understanding of the environment, and nuclear issues are too important to be left to specialists. The more the traditional disciplines become engaged in the study and teaching of the questions that link science, technology and international affairs, the better informed will be our approaches to these thorny yet critical issues. Until this becomes the accepted approach, it is essential that programs like STIA provide an intellectual home where this important dimension of international affairs can receive the attention it requires and deserves.

## Footnotes

1 A separate paper sets forth a half a dozen basic patterns of interaction that characterize a broad range of science- and technology-intensive issues on the current international agenda. See C. Weiss, “What Holds Science, Technology and International Affairs (STIA) Together as an Academic Discipline?,” paper presented at the Penn State Conference on “The Evolving Role of Science and Technology in Foreign Relations: Implications for International Affairs in the 21^st^ Century,” October 23, 2009.

